# Cardiac Potassium Channels: Physiological Insights for Targeted Therapy

**DOI:** 10.1177/1074248417729880

**Published:** 2017-09-25

**Authors:** Kamalan Jeevaratnam, Karan R. Chadda, Christopher L.-H. Huang, A. John Camm

**Affiliations:** 1 Faculty of Health and Medical Sciences, University of Surrey, Guildford, United Kingdom; 2 School of Medicine, Perdana University–Royal College of Surgeons Ireland, Serdang, Selangor Darul Ehsan, Malaysia; 3 Physiological Laboratory, University of Cambridge, Cambridge, United Kingdom; 4 Division of Cardiovascular Biology, Department of Biochemistry, University of Cambridge, Cambridge, United Kingdom; 5 Cardiac Clinical Academic Group, St George’s Hospital Medical School, University of London, Cranmer Terrace, London, United Kingdom

**Keywords:** potassium channels, repolarization, physiological mechanisms, currents, ion channel, drug target

## Abstract

The development of novel drugs specifically directed at the ion channels underlying particular features of cardiac action potential (AP) initiation, recovery, and refractoriness would contribute to an optimized approach to antiarrhythmic therapy that minimizes potential cardiac and extracardiac toxicity. Of these, K^+^ channels contribute numerous and diverse currents with specific actions on different phases in the time course of AP repolarization. These features and their site-specific distribution make particular K^+^ channel types attractive therapeutic targets for the development of pharmacological agents attempting antiarrhythmic therapy in conditions such as atrial fibrillation. However, progress in the development of such temporally and spatially selective antiarrhythmic drugs against particular ion channels has been relatively limited, particularly in view of our incomplete understanding of the complex physiological roles and interactions of the various ionic currents. This review summarizes the physiological properties of the main cardiac potassium channels and the way in which they modulate cardiac electrical activity and then critiques a number of available potential antiarrhythmic drugs directed at them.

## Introduction

**Figure 1.**
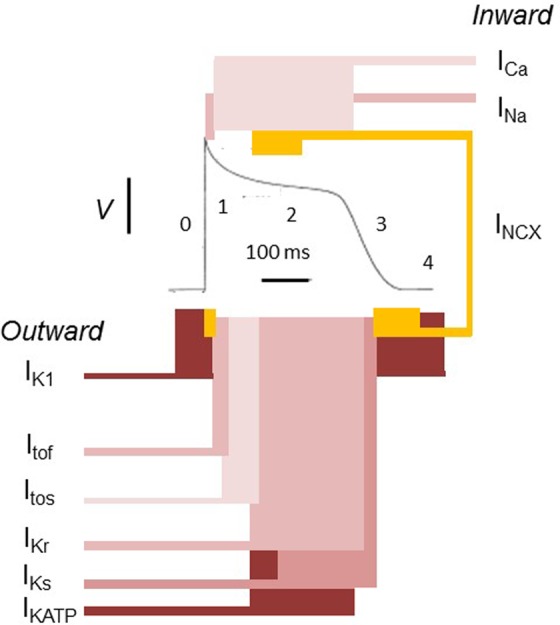
The ventricular action potential as a paradigm for cardiac electrophysiological activity. In the resting state, the voltage of the cell intracellular space is negative to the external environment. This reflects its higher K^+^ but lower Na^+^ and Ca^2+^ concentrations and its lower membrane permeability to Na^+^ and Ca^2+^ in comparison to K^+^. K^+^ efflux from the cell is then controlled by the inward rectifier K^+^ channel (*I*_K1_). When excitation threshold is reached, a large Na^+^ influx (*I*_Na_) into the cell through Na^+^ channels produces phase 0 depolarization. This is followed by activation of fast and slow transient outward K^+^ currents (*I*_tof_ and *I*_tos_, respectively) mediating a K^+^ efflux driving a rapid phase 1 repolarization. There is also an activation of a depolarizing inward Ca^2+^ current through L-type Ca^2+^ channels (*I*_Ca_), which initiates excitation contraction coupling. The reduced membrane K^+^ permeability due to *I*_K1_ rectification combined with *I*_Ca_ maintains the action potential phase 2 plateau phase. Phase 3 repolarization is driven by K^+^ efflux through the rapid and slow delayed rectifier K^+^ channels (*I*_Kr_ and *I*_Ks_, respectively), as well as *I*_K1_. At the end of phase 3, the Na^+^ and Ca^2+^ that have accumulated in the cells are removed by the Na^+^, K^+^ pump, and the Na^+^, Ca^2+^ exchanger (NCX). The atrial action potential shows greater contributions to recovery from the ultrarapid delayed rectifier outward currents (*I*_Kur_) and acetylcholine-activated inward rectifying K^+^ channel (*I*_KACH_). Adapted with permission from Huang.^1^

Orderly propagation of cardiac electrophysiological excitation and recovery depends on a normal sequence of cardiac action potential (AP) generation through its component myocytes. The depolarization and repolarization of AP is mediated by multiple, interacting, inward and outward currents mediated by different ion charge carriers dependent on the action of specific membrane ion channels (Figure 1). The initial depolarization phase takes the form of a rapid upstroke and is mainly driven by inward Na^+^ current (*I*_Na_) through voltage-gated sodium channels (Na_v_1.5). The succeeding plateau phase is dominated by inward Ca^2+^ current (*I*_Ca_). The resulting entry of extracellular Ca^2+^ induces release of sarcoplasmic reticular Ca^2+^ stores, thereby activating excitation–contraction coupling. Repolarization, ultimately returning the membrane to the resting potential, is principally driven by outward current through voltage-gated K^+^ channels (K_v_).^1^ K^+^ channel activity is thus a principal determinant of AP duration (APD) as it limits the depolarization duration and therefore both the time course of the Ca^2+^-mediated contraction and the refractory period. There are numerous and diverse K^+^ channels types, each with particular kinetic and voltage-dependent properties. These result in numerous and diverse current contributions, each with specific roles at different phases of repolarization. Together these determine the relatively prolonged but finely tuned repolarization time course and the repolarization reserve following recovery of the resting membrane potential. The repolarization reserve refers to the partly overlapping function of these currents, namely, *I*_Kr_, *I*_Ks_, and *I*_K1_, that gives a limited level of redundancy to the system.^2^ The kinetics of repolarization varies greatly with cardiac region and species. This reflects variations in the occurrence and density of the different K^+^ channel subtypes. All these characteristics suggest that explorations of K^+^ channels may yield a useful group of pharmacological targets for arrhythmic conditions.

### Potassium Channels

**Figure 2.**
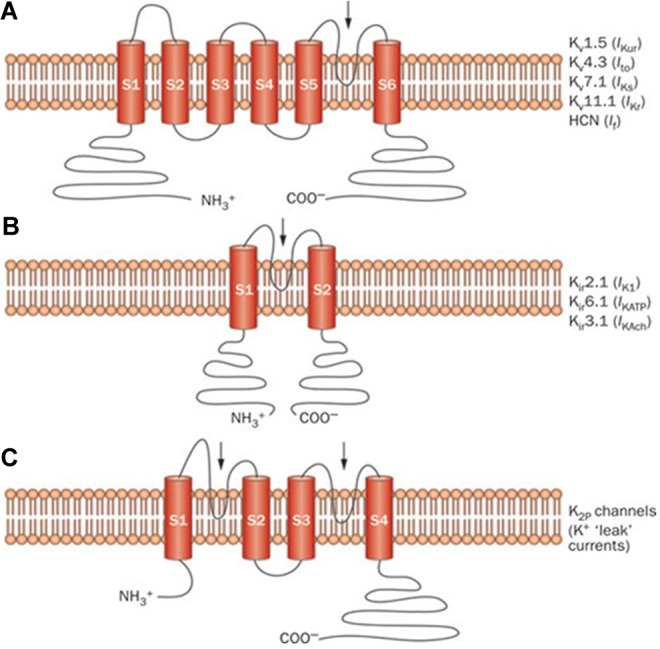
Structure of different cardiac potassium channel species: Schematic representation of selected potassium channel α-subunits. A, The 6-transmembrane 1-pore-region voltage-dependent K^+^ channel (K_v_) α-subunits mediating *I*_Kur_, *I*_to_, *I*_Ks_, *I*_Kr_, and *I*_f_. B, The 2-transmembrane 1-pore-region inward rectifying K^+^ channel (*K*_ir_) α-subunits mediating *I*_K1_, *I*_KATP_, and *I*_KAch_. C, The 4-transmembrane 2-pore-region K^+^ channel (K_2P_) mediating “leak” K^+^ currents. The arrows indicate the location of the pore-forming region(s). HCN indicates hyperpolarization-activated cyclic nucleotide-gated channel; *I*_f_, inward rectifier mixed Na^+^ and K^+^ “funny” current; *I*_K1_, inward rectifier K^+^ current; *I*_KACH_, acetylcholine-activated inward rectifier K^+^ current; *I*_KATP_, ATP-sensitive K^+^ current; *I*_Kr_, rapid component of the delayed rectifier K^+^ current; *I*_Ks_, slow component of the delayed rectifier K^+^ current; *I*_Kur_, ultrarapid component of the delayed rectifier K^+^ current; *I*_to_, transient outward K^+^ current. Reprinted with permission from Giudicessi and Ackerman. Macmillan Publishers Ltd, copyright 2012.^3^

K^+^ channels represent the most functionally diverse cardiac ion channel type.^3-6^ Together, they tightly regulate cardiac repolarization, thus ensuring stable and consistent AP signaling. The different K^+^ channel types have overlapping functions,^2,7^ resulting in some degree of functional redundancy,^2^ which in turn contributes to repolarization reserve. Table 1 summarizes their encoding genes with their chromosomal locations and the structural properties of their pore-forming α- and accessory β-subunits. The α-subunit of different K^+^ channel types all possess a conserved pore-forming region allowing K^+^ movement across the plasma membrane down an electrochemical gradient possessing a selective permeability to K^+^ attributable to a specific structural motif. They may also exhibit gating mechanisms responsive to membrane depolarization and ligand-binding sites whose occupancy could alter channel conformation. Finally, individual monomeric α-subunits may assemble into functional dimers or tetramers due to the presence of one or more subunit-assembly domains.^6,8-10^ K^+^ channel α-subunits fall into 3 structural types based on subunit topology (Figure 2). The first has 1 pore-forming region with 6 or 7 transmembrane regions (Figure 2A), the second has 1 pore-forming region and 2 transmembrane regions (Figure 2B), and the third has 2 pore-forming and 4 transmembrane regions (Figure 2C).^5,6,10^

**Table 1.** Molecular Details and Activation Mechanisms of the Cardiac Potassium Channels.^2^

Current	Gene	Chromosomal Location	Associated Protein	Type of Subunit
*I*_tof_	*KCND3*	1p13.2	K_v_4.3	α
	*KCNIP2*	10q24.32	KChIP2	β
	*KCNE3*	11q13.4	MiRP2	β
*I*_tos_	*KCNA4*	11p14.1	K_v_1.4	α
*I*_Ks_	*KCNQ1*	11p15.5-p15.4	K_v_1.7.1/K_v_LQT1	α
	*KCNE1*	21q22.12	minK	β
	*AKAP9*	7q21.2	AKAP-9	β
*I*_Kr_	*KCNH2*	7q36.1	K_v_11.1/hERG	α
	*KCNE2*	21q22.11	MiRP1	β
*I*_K1_	*KCNJ2*	17q24.3	K_ir_2.1/IRK1	α
	*KCNJ12*	17p11.2	K_ir_2.2/IRK2	α
*I*_KATP_	*KCNJ8*	12p12.1	K_ir_6.1	α
	*KCNJ11*	11p15.1	K_ir_6.2	α
	*ABCC9*	12p12.1	SUR2A/SUR2B^a^	β
*I*_Kur_	*KCNA5*	12p12.32	K_v_1.5	α
	*KCNAB1-B3*	N/A	K_v_β1-3	β
*I*_KAch_	*KCNJ3*	2q24.1	K_ir_3.1/GIRK1	α
	*KCNJ5*	11q24.3	K_ir_3.4/GIRK4	α

Abbreviations: *I*_K1_, inward rectifier K^+^ current; *I*_KACH_, acetylcholine-activated inward-rectifier K^+^ current; *I*_KATP_, ATP-sensitive K^+^ current; *I*_Kr_, rapid component of the delayed rectifier K^+^ current; *I*_Ks_, slow component of the delayed rectifier K^+^ current; *I*_Kur_, ultrarapid component of the delayed rectifier K^+^ current; *I*_tof_, fast transient outward K^+^ current; *I*_tos_, slow transient outward K^+^ current.

^a^SUR2A and SUR2B are splice variant of ABCC9 and considered as cardiac (SUR2A) and vascular (SUR2B) isoforms.

K^+^ channel β-subunits encompass many molecular groups, such as adenosine triphosphate (ATP)–binding cassette transport-related proteins (eg, sulfonylurea receptors) for inward rectifiers, cytoplasmic proteins (KChIP, KChAP, and K_v_β1-3), and single transmembrane spanning proteins (minK).^10^ These β-subunits form complexes with the α-subunits and can modify the channel’s functional properties. For example, K_v_β subunits can alter channel trafficking and the kinetics of current activation and inactivation when interacting with K_v_1.5.^11^ More specifically, K_v_β2.1 and K_v_β4.1 behave as chaperone proteins.^12^ Furthermore, the N-terminus of K_v_β1.2 and K_v_β1.3 has an inactivation domain resembling the inactivation particle of the α-subunit, allowing it to modulate 2channel inactivation.^12-14^

### Cardiac Potassium Currents

**Figure 3.**
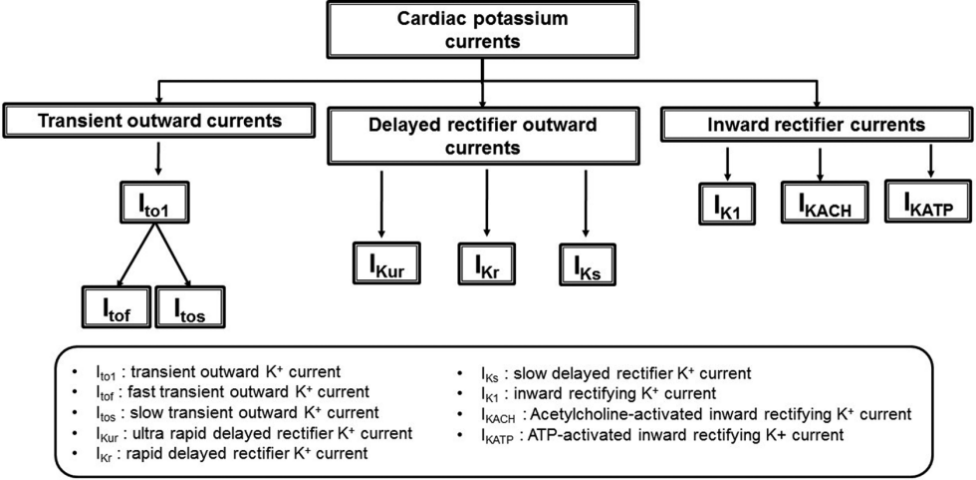
Classification of K^+^ currents: General classification of the main cardiac K^+^ currents. The relatively new additions to the K^+^ channel family (*Ca^2+^-activated K^+^ current [I*_KCa_*]* and *2-pore domain K^+^ current [I*_K2p_*]*) have not been grouped under this scheme. Most K^+^ currents are grouped according to the direction of their overall rectification property. In some instances, this may vary. With the inward rectifying K^+^ channels, the name refers to the unusual characteristic whereby net potassium flow is into the cell at potentials lower than the reversal potential where channel conductance is high. As the channel potential becomes more positive, channel conductance decreases. Net ion flow direction reverses at the reversal potential, meaning that net potassium flow is outward at potentials more positive than this. Therefore, at depolarized potentials, potassium loss from the cell is low as conductance through the channel is low.

Cardiac K^+^ channels vary in their permeability properties, membrane potential dependence, and their opening or closing activation and inactivation kinetics. The major currents are classified into the transient outward currents, delayed rectifier outward currents, and the inward rectifiers (Figure 3). Advances in electrophysiological and molecular biology techniques have demonstrated additional currents that may fall outside this basic classification. Some brief notes on the major cardiac K^+^ currents, their role in the cardiac AP, and their functional importance follow.

#### Transient outward K^+^ (I_to1_) currents

When first described, the transient outward currents (*I*_to_) were attributed to 2 distinct channels, one blocked by 4-aminopyridine (4-AP) and unaffected by extracellular Ca^2+^ (*I*_to1_) and the other not blocked by 4-AP but sensitive to Ca^2+^ (*I*_to2_).^6^ *I*_to_ drives the initial rapid repolarization phase of the AP. Regions with shorter APDs, such as the epicardium, right ventricle, and septum, have higher *I*_to_ expression. It was later discovered that *I*_to2_ is a Cl^-^ rather than a K^+^ current.^15^ Further characterizations subdivided *I*_to1_ into fast (*I*_tof_) and slow (*I*_tos_) currents (Figure 3). *I*_tof_ predominates in the atria, whereas both *I*_tof_ and *I*_tos_ occur in the ventricles.^16^ While *I*_tos_ requires longer recovery times, its classification as “slow” is relative only to *I*_tof_. Thus, both *I*_tof_ and *I*_tos_ channels activate and inactivate rapidly in comparison to the corresponding processes in other K^+^ channels.^15^ Due to differences in the biophysical properties of *I*_tof_ and *I*_tos_, the existence of molecular heterogeneity between these 2 channels has been previously suggested.^15^

#### Ultrarapid delayed rectifier currents (I_Kur_)

In addition to *I*_to_, the ultrarapid delayed rectified K^+^ current (*I*_Kur_) plays a role in the initial rapid phase 1 AP repolarization. *I*_Kur_ activates rapidly in under 10 milliseconds at voltages in the plateau range and deactivates slowly over the course of the AP.^17-19^ *I*_Kur_ is the predominant delayed rectifier current for the atria and thus results in the shorter APD seen in the atria compared to the ventricles.^10,16,17,19^ Where *I*_Kur_ is present, its channels are not evenly distributed over the myocyte surface but instead found at high densities in the intercalated disk.^6^ This pattern of distribution is often disrupted after cardiac ischemic damage.^10^ The selective presence of *I*_Kur_ in the atria makes it an interesting target for atria selective therapy, whereby inhibition of *I*_Kur_ would prolong the APD in the atria but not the ventricles.^4^

#### Rapid delayed rectifier K^+^ currents (I_Kr_)

The voltage-gated rapid delayed rectifier outward K^+^ current (*I*_Kr_) is critical to phase 3 repolarization. It shows a relatively rapid activation with depolarization. However, its inactivation rate is around 10 times faster than its activation rate due to voltage-dependent C-type inactivation. This renders it relatively nonconducting in phases 1 and 2 of the cardiac AP.^20-23^ Thus, although termed a delayed rectifier current, it also shows an inward rectification property at positive potentials.^22,24^ However, with the end of phases 1 and 2, as the membrane potential becomes negative to 0 mV, *I*_Kr_ becomes activated once again, but the deactivation during this phase is much slower. This results in a large outward K^+^ efflux during phase 3 repolarization.^2,10^ *I*_Kr_ is found in both human atria and ventricles but is differentially expressed with higher levels in the left atrium and ventricular endocardium.^16^

#### Slowly activating delayed rectifier K^+^ current (IKs)

Cardiac repolarization is also influenced by a third, slowly activating delayed rectifier K^+^ current (*I*_Ks_). *I*_Ks_ slowly activates at potentials positive to −20 mV. Unlike *I*_Kr_, *I*_Ks_ barely inactivates^25,26^ and consequently accumulates over phase 2 repolarization, significantly influencing phase 3 repolarization.^2^ This feature of *I*_Ks_ is particularly important during atrial and ventricular APs of long duration. It is also involved in APD shortening during physiological increases in heart rate. An increase in heart rate thus reduces the time required for *I*_Ks_ inactivation. In consequence, more *I*_Ks_ accumulates, leading to a steeper drop in the repolarization rate.^27,28^ Blocking *I*_Ks_ results in an APD prolongation at increased heart rates.^21,28^ Inhibition of *I*_Ks_ will increase the vulnerable window for reactivation of voltage-gated Ca^2+^ channels, thereby increasing the risk of arrhythmic trigger events.^2^ *I*_Ks_ is found in all cardiac cell types, but its expression is significantly reduced in the mid-myocardial wall; this accounts for the long APD seen in this region.^16^

#### Inward rectifier K^+^ current (I_K1_) 

The inward rectifier K^+^ current (*I*_K1_) functions over a narrow membrane potential range. Its rectifying property results in a marked reduction in *I*_K1_ conductance at positive, depolarized, membrane potentials and an increase in *I*_K1_ at negative membrane potentials, with the effect of stabilizing the membrane resting potential close to the K^+^ equilibrium potential (*E*_K_).^10^ The channel mediating *I*_K1_ does not show voltage-dependent gating and does not possess a voltage sensor. Nevertheless, *I*_K1_ modulation associated with movement of Mg^2+^ and polyamines results in an indirect sensitivity to voltage.^29-32^ Between phase 0 and phase 2 of the AP, the membrane potential is more positive than −20 mV, and at this potential, there is no conductance of *I*_K1_ as the channel is inhibited by Mg^2+^ and polyamines. The resulting marked inward rectification property limits the outward current at these positive potentials. This in turn minimizes the inward depolarizing current, which confers energetic efficiency for AP generation as it minimizes changes to ionic gradients that would need to be restored.^16^ As the potential returns to more negative values (typically around −40 mV), the inhibition by Mg^2+^ and polyamines is reversed. *I*_K1_ conductance then resumes and this contributes to phase 3 cardiac repolarization.^31^ *I*_K1_ occurs in both atria and ventricles and is thereby involved in setting their resting membrane potentials. Channels conducting *I*_K1_ are expressed in greater density in the ventricles, making the ventricles less susceptible to pacemaker influence.^16^

#### Acetylcholine-activated K^+^ current (I_KACH_)

The inwardly rectifying acetylcholine (ACh)-activated K^+^ current (*I*_KACH_) is regulated by G proteins rather than voltage gating. Cardiac parasympathetic nerve endings release ACh, thereby activating M2 muscarinic receptors. This reduces the depolarizing effect of the pacemaker current (*I*_f_), reducing firing rates of pacemaker cells and in turn reducing heart rate.^6^ Acetylcholine also opens muscarinic-sensitive *I*_KACH_ channels allowing the inward rectification of K^+^. The inward rectifying current shortens the AP and hyperpolarizes the membrane potential.^16^ Membrane hyperpolarization reduces the rate at which the sinoatrial and atrioventricular (AV) nodes drive pacemaker depolarization in addition to reducing AV conduction velocity.^6,33^ *I*_KACH_ is thought to be specific to the atria,^2^ but there has been a suggestion that it may exist both in the atria and ventricle,^16^ but with densities 6 times greater in the atria than the ventricles.^34^

#### ATP-activated K^+^ current (I_KATP_)

The ATP-activated K^+^ current (*I*_KATP_) occurs at both the sarcolemmal (sarc-K_ATP_) and mitochondrial inner membrane (mito-K_ATP_) of cardiomyocytes. The sarc-K_ATP_ channels are highly expressed in cardiomyocytes and are composed of K_ir_6.2 and SUR2A subunits. There may also be contributions from K_ir_6.1 and SUR1.^35^ In contrast, although the subunits of mito-K_ATP_ channels have been difficult to identify due to the challenge of isolating pure mitochondrial membrane fractions, ROMK2 pore-forming subunits and SUR2 regulatory subunits have been suggested to contribute.^36,37^

Both channels are controlled by ATP and are thus directly responsive to the cell’s metabolic status, thereby influencing cell membrane potential.^6^ *I*_KATP_ is inhibited by physiological intracellular ATP levels, but this reverses with ATP depletion. Thus, under normal energetic circumstances, there is limited *I*_KATP_ current. However, under both physiological and pathological conditions that reduce ATP, there is increased *I*_KATP_ current that is essential for adaptation to stress. For example, compared to wild-type controls, mice lacking K_ir_6.2-containing K_ATP_ channels perform less well in acute treadmill exercise testing.^38^ The increased *I*_KATP_ has a cardioprotective role in ischemia by shortening the cardiac AP, thus limiting calcium influx into the cytosol.^39-41^ Specifically, studies have suggested that mito-K_ATP_ rather than sarc-K_ATP_ channel opening has an energy-modulating property that confers cardioprotection in ischemic hearts.^42,43^

In some situations, the *I*_KATP_-mediated APD shortening and corresponding heterogeneities in repolarization can create a substrate for cardiac reentry arrhythmia. In other situations, K_ATP_ channel openers have been described to have antiarrhythmic effects,^44-48^ and evidence suggests that activation and block of K_ATP_ can be pro- or antiarrhythmic depending on the arrhythmogenic mechanism in different animal models.^49^ For example, selective sarcolemma K_ATP_ channel blockers, such as HMR 1883, confer antiarrhythmic effects in the short term,^50^ although this could be metabolically disadvantageous in the long term due to the abolished adaptive response to stresses. Finally, it is important to note that the channel involved in the conductance of *I*_KATP_ is also thought to be involved in the regulation of smooth muscle tone and insulin secretion in pancreatic β-cells.^6^

#### Other K^+^ channel family: Ca^2+^-activated K^+^ current (I_KCa_), 2-pore domain K^+^ current (I_K2p_), and hyperpolarization-activated cyclic nucleotide-gated channels

Recently, several further currents have been characterized. The Ca^2+^-activated K^+^ current, also known as the small conductance Ca^2+^-activated K^+^ (SK) current (*I*_KCa_), and the 2-pore domain K^+^ current (*I*_K2p_) have attracted considerable physiological and pharmacological interest. *I*_KCa_ was initially thought to not exist in the human heart.^51^ However, subsequent studies demonstrated the presence of *I*_KCa_, with a higher density in the atria than the ventricle. Various subtypes of Ca^2+^-activated K^+^ channels exist in different tissues; the channel subtype conducting the cardiac *I*_KCa_ is the SK channel.^51,52^ In neuronal cell, SK channels that are involved in modulating the tonic firing frequency and activation of these channels cause membrane hyperpolarization, thus limiting neuronal AP firing frequency.^51^ In contrast, cardiac SK channels and consequently *I*_KCa_ are involved in late AP repolarization, controlling the resting membrane potential in human atria.^52^ *I*_KCa_ appears to not play physiologically significant roles in the ventricle.^52^ *I*_KCa_ is accordingly of particular pharmacological interest for atrial fibrillation (AF) therapy. Thus, *I*_KCa_ occurs during late repolarization, when the atrial AP is susceptible to irregular or abnormal excitation such as that resulting from early after-depolarizations (EADs).^51^

*I*_K2p_ contributes to the background current, the resting membrane potential, and cellular excitability. The channel involved in the conductance of this current has no voltage dependence, but its activity is modulated by lipids, particularly fatty acids, pH, drugs, particularly local and inhalation anesthetics, and membrane stretch.^53,54^ These mediators act upon the channel via secondary messenger phosphorylation.^55^ *I*_K2p_ is a background current that persists through all phases of the cardiac AP. It thus stabilizes the membrane potential toward *E*_m_. *I*_K2p_ may also prevent the occurrence of EADs, and it may be involved in fine-tuning of Na^+^ channel availability for phase 0 depolarization.^56^ The current has been found to occur selectively in the atria and AV node, thereby making it a target for drug development.^57-59^ Although not entirely new but only recently well characterized, the hyperpolarization-activated cyclic nucleotide-gated (HCN) channel is instrumental in conducting the inward funny current (*I*_f_) in the heart. The channel is activated by the hyperpolarization of the membrane and is additionally stimulated by intracellular cyclic nucleotides.^60,61^ The generation of *I*_f_ is attributable to the inward permeability of both Na^+^ and K^+^ and occurs at threshold close to the resting membrane potential.^62^ Although the HCN channel under physiological circumstances conducts both Na^+^ and K^+^, the primary sequence of the HCN pore region suggests that it is primarily related to a selective potassium channel.^63^ In certain pathological conditions such as AF and myocardial infarction, *I*_f_ is increased unusually outside the pacemaker cells, leading to increased propensity to arrhythmia. Thus, targeting the *I*_f_ in such pathological conditions has proven to be therapeutically advantageous.^64^

## Cardiac K^+^ Channel as Targets for Drug Development

Although there have been significant recent advances in the development and use of cardiological devices and procedures directed at arrhythmic conditions, antiarrhythmic drugs continue to be important whether by themselves or as adjunct therapy to such interventions. These include situations involving acute management of potentially fatal arrhythmic events, particularly where such procedures are contraindicated. Yet progress in antiarrhythmic drug development has been relatively limited. This likely reflects a lack of understanding of cardiac arrhythmic mechanisms. However, recent developments of our understanding of the role of the ion channels in normal AP generation have led to a specific interest into ion channels and their associated currents whose abnormal activity potentially leads to arrhythmia. This would encourage interest in the development of cardiac ion channel activator or blockers directed at modulating the cardiac AP or its refractory period. Introduction of drugs acting specifically on ion channels would optimize the efficacy of therapeutic actions on arrhythmogenic tendency, while minimizing problems arising from potential cardiac and extracardiac toxicity. K^+^ channels play a vital role in cardiac AP repolarization and thus naturally form potential targets for the development of ion channel-specific antiarrhythmic therapy, such as for AF. However, a limitation of this approach is that arrhythmic conditions, such as AF, are heterogeneous and the efficacy of targeting ion channels varies according to the cause and extent of the arrhythmia.

This is complicated by the fact that in various physiological and pathological conditions, remodeling of K^+^ channel expression can occur, which can alter the AP and increase the risk of sudden cardiac death.^65^ For example, AF is maintained and progressed partly due to electrical remodeling, mediating APD shortening.^66^ Thus, in chronic AF, there is upregulation of *I*_K1_, *I*_Ks_, and *I*_K2P_3.1, which offsets the possible downregulation of *I*_Kur_ and *I*_to_.^67-69^ Nevertheless, the experimental evidence for the reduction in *I*_Kur_ during remodeling is conflicting, as some reports suggest reduced *I*_Kur_ density^70,71^ and others suggest no change.^72,73^ It has been suggested that receptor-activated *I*_KACH_ (r*I*_KACH_) mediates AF induced by vagal stimulation, while constitutive *I*_KACH_ (c *I*_KACH_) develops in the time course of AF remodeling.^67,74^

In physiological cardiac hypertrophy, induced by chronic exercise, for example, there is an increase in *I*_K_ density.^75^ This contrasts with pathological cardiac hypertrophy caused by pressure overload where a reduced *I*_K_ density is noted that was attributable to cellular hypertrophy rather than gene expression changes in *I*_tof_ and *I*_K1_.^76^ In heart failure, AP prolongation is associated with downregulation of several genes, leading to reduced *I*_tof_, *I*_Ks_, *I*_Kr_, and *I*_K1_.^65,77,78^ Considering the changes in K^+^ channel expression in remodeling is clinically important as the sensitivity and efficacy of blocking these channels will change.

Table 2 outlines selected drugs that have been experimentally proven to target different K^+^ channels, using either native cardiac myocytes or human cell line expression systems. Some of these drugs presently in clinical use have been primarily developed for other ion channels such as the Na^+^ or Ca^2+^ cardiac ion channel but have corresponding effects on K^+^ channels. Several drugs have been proposed to be selective to specific K^+^ channels, such as A935142, XEN-D0103, and XEN-D0101. However, despite promising experimental findings, many of these drugs have not progressed to clinical use. This may be attributable to limitations associated with experimental studies. Expression systems can often produce off-target effects or nonspecific interactions which may mask the true effect of these drugs. Additionally, expression systems may run the risk of either overexpressing or underexpressing the channel of interest. On the other hand, native cardiac myocytes, while more physiologically representative, may not provide the right platform for the study of specific targets. Additionally, acquisition of viable native cardiac myocytes from a minimally heterogeneous population remains a challenge, and it is widely accepted that channel functions can differ by gender and age. Consequently, while experimental studies may suggest potentially promising options to selectively target K^+^ channels, the translational capacity of such studies remains limited.

**Table 2.** Selected Pharmacological Agents Affecting the Human K^+^ Channels.

Current	Pharmacological Agent (Expression System), Reference
Activators	
* I*_Kr_	A-935142 (HEK),^79^ ICA-105574 (HEK),^80^ NS1643 (HEK),^81^ PD-118057 (HEK)^82^
* I*_Ks_	Ephedrine (HEK),^83^ Tanshinone IIA (HEK)^84^
* I*_Kca_	NS1619 (HEK)^85^
Blockers	
* I*_to_	Chromanol 293B (nHVM),^86^ Flecainide^a^ (nHAM)^87^
* I*_Kur_	Amiodarone^a^ (HEK),^88^ Bepridil (HEK),^88^ DPO-1 (nHAM),^89^ MK-0448 (nHAM),^90^ NIP-142 (HEK),^91^ Papaverine (nHAM),^92^ Pimozide (HEK),^93^ Sertindole (HEK),^94^ XEN-D0103 (nHAM)^95^
* I*_Kr_	Cocaine (HEK),^96^ Fluvoxamine (HEK),^97^ Ketoconazole (HEK),^98^ Ketanserin (HEK),^99^ Ziprasidone (HEK)^100^
* I*_Ks_	HMR 1556 (HEK),^101^ SKF-96365 (HEK)^102^
* I*_KACH_	NIP-151 (HEK),^103^ U73122/U73343 (HEK)^104^
* I*_KATP_	5-Hydroxydecanoate (HEK),^105^ HMR1098 (HEK)^105^
* I*_to_, *I*_Kur_	AVE-0118 (nHAM),^106^ Acacetin (nHAM),^107^ Ambasilide (nHAM),^108,109^ 4-aminopyridine (nHAM),^87^ Diltiazem^b^ (nHAM),^110^ Docosahexaenoic acid (nHAM),^111^ Eicosapentaenoic acid (nHAM),^111^ Nifedipine^b^ (nHAM),^110^ Quinidine^a^ (nHAM),^87^ Raloxifene (nHAM),^112^ U50488 H (nHAM),^113^ XEN-D0101 (nHAM),^114^ Vernakalant (RSD1235) (HEK)^115^
* I*_to_, *I*_Kur_, *I*_K1_	Propafenone^a^ (nHAM)^116^
* I*_to_, *I*_Kur_, *I*_Kr_, *I*_Ks_	Clotrimizole (nHAM)^117^
* I*_to_, *I*_Kur_, *I*_Kr_, *I*_Ks_, *I*_K1_	Azimilide (nHAM)^118^
* I*_Kur_, *I*_Kr_	Cisapride (HEK),^119^ Verapamil^b^ (nHAM, HEK)^120,121^
* I*_Kr_, *I*_Ks_	Sotalol (nHAM)^21,108^

Abbreviations: nHAM, native human atrial myocyte; HEK, human embryonic kidney; nHVM, native human ventricular myocyte; *I*_K1_, inward rectifier K^+^ current; *I*_Kr_, rapid component of the delayed rectifier K^+^ current; *I*_KACH_, acetylcholine-activated inward rectifier K^+^ current; *I*_KATP_, ATP-sensitive K^+^ current;; *I*_Ks_, slow component of the delayed rectifier K^+^ current; *I*_Kur_, ultrarapid component of the delayed rectifier K^+^ current; *I*_to_, transient outward K^+^ current; *I*_KCa_, small conductance Ca^2+^-activated K^+^ (SK) current.

^a^Primary Na^+^ channel blocker.

^b^Primary Ca^2+^ channel blocker.

Furthermore, these activators and blockers often target more than 1 K^+^ channel species and thus are not entirely specific.^10,122^ However, a large proportion of these drugs also typically target *I*_Kr_ (known to be present in all cardiac regions) and as such do not constitute ideal candidates for targeted therapy. Nevertheless, mechanisms of cardiac arrhythmia are likely to be region dependent. Drugs that may be antiarrhythmic in some cardiac regions may potentially be pro-arrhythmic in others. Thus, the presence of atrial-specific K^+^ channels has provided focus on developing drugs that could specifically increase refractory periods, thus preventing atrial reentry arrhythmia, which is the most common mechanism for AF.^123^

Of ion channels specific to the atrium that might offer specific therapeutic targets, the channel conducting *I*_Kur_ tends to prolong repolarization and effective refractory period (ERP) without altering QT intervals.^124,125^ The experimental drugs AVE0118 and XEN-D101 are thought to be *I*_Kur_ selective blockers with both prolonging APD in atrial tissue from patients with permanent AF in common with the known *I*_Kur_ blocker 4-AP.^106-129^ However, a subsequent “first-in-human” study using the highly selective *I*_Kur_ blocker MK-0448 (N-{6-[(1S)-1-(4-fluorophenyl)-2,2-di (pyridine-3-1) ethyl] pyridine2yl} methane sulfonamide) did not reveal any increase in atrial ERP. This led to the conclusion that selective blocking of *I*_Kur_ may have limited clinical value.^130^ *I*_KACH_ channels are also atrium specific or at least predominantly occur in the atria and have minimal physiological function in the ventricle.^129^ Opening of the *I*_KACH_ channel will lead to shortening of atrial APD and thus increase the likelihood of AF. Therefore, blocking the opening of *I*_KACH_ channels will prevent such shortening of APD with minimal effect on ventricular APD, in turn reducing the chances of AF. Several drugs block *I*_KACH_, but have limited specificity. Nevertheless, selective blocking of *I*_KACH_ has been experimentally achieved using the compound NTC-801. The compound was found to have selective antifibrillatory properties, achieved by prolonging the atrial ERP.^131^ Another potential atrial-specific therapeutic target of interest is the *I*_KCa_ current conducted by SK channels. The selective presence of this current in the atria has recently led to several investigative drugs beings explored. NS8593 is a selective SK channel inhibitor demonstrating significant atrial antiarrhythmic effects in canine and equine experimental models. Experiments using human atrial cardiac myocytes from patients with normal sinus rhythm demonstrated reduction in K^+^ currents and prolongation in APD. No such changes were observed in intraventricular myocytes.^52^

## Conclusion

There is currently an incomplete understanding of the cellular physiological role of the various cardiac potassium currents and their interacting effects and how dysregulation of their function and expression can provide arrhythmogenic mechanisms. It is thought that the site-specific distribution of some K^+^ channels could allow targeted therapy to be more spatially selective. However, complex electrical remodeling events that occur in disease states may change channel expression levels to the extent that the selectivity of the drug is hindered, making even this potential therapeutic strategy challenging. Although targeting ion channels responsible for discrete parts of the cardiac AP to modulate the system towards a more physiological state has therapeutic appeal, there are inherent difficulties in developing successful drugs. This is because the ion channels targeted are functionally complex and are interdependent, thus adding a dynamic situation in which function and expression are altered depending on the cell environment. Furthermore, pathophysiological processes of arrhythmic disease may involve functional alterations in 1 or more ion channels. Such single or multiple ion channel functional abnormalities may therefore warrant corresponding use of a single or multichannel activator/blocker approach. However, this approach will only be possible if we are able to identify the specific pathophysiological process affecting individual patients (ie, is this arrhythmic disease related to a single or multichannel abnormality). Thus, although we may be able to develop single or multichannel activators/blockers, actual clinical use will be dependent on a detailed understanding of the exact arrhythmogenic mechanisms affecting individual patients, which thus far is limited. Presently, decisions to use single or multichannel activators/blockers are largely dependent on resolution of clinical signs or the actual arrhythmia rather than a therapeutic approach targeting ion channel functional abnormality. Furthermore, the availability of truly specific ion channel activators/blockers is limited as these agents tend to have off-target actions with corresponding side effects, and this limits the clinical use of selective agents. Focusing on understanding the system at a cellular physiological level through further experimental and computational modeling is needed to enable development of novel insights at a pharmacological level.
